# The spider model for clinical involvement in radiology

**DOI:** 10.1007/s13244-014-0326-4

**Published:** 2014-05-21

**Authors:** Jim A. Reekers

**Affiliations:** Academic medical Center Amsterdam, Department of Radiology, Meibergdreef 9, Amsterdam, 1105 AZ The Netherlands

## Abstract

How to stay in the driver’s seat in the future as a radiologist: subspecialisation and direct clinical involvement are crucial to remain of added value to the hospital.

*Teaching Points*

- *Subspecialisation and clinical involvement are crucial for future radiologists.*
- *Visibility of the radiologist is important.*
- *The European Society of Radiology (ESR) should coordinate subspecialisation with teaching and accreditation.*

Radiology has always been categorised as a medical specialty that mainly provides support to clinicians. There are only a few other medical specialties that are also seen as supportive, such as pathology and anaesthesiology. However, these other specialties have always had a completely different position within the hospital environment compared with radiology. They have 100 % direct contact to patients or patient-related specimens and they also have a specialist knowledge which is undisputed and recognised as unique. No clinician will try to read tissue specimens or perform his own general sedation; apart from the fact that this would lead to a serious malpractice case, it is just not done. However, a clinician reading radiological images is very common and sometimes the reporting by the radiologist is only an economic formality. In 2009, Dr. Rafel Tappouni presented a survey, held in his own hospital at the Radiological Society of North America (RSNA) about how clinicians deal with radiology reports [1]. More than 90 % of the respondents said they were comfortable interpreting X-rays; in 55.3 % all of the time and 35.8 % some of the time. More than half said they were equally competent at interpreting computed tomography (CT) exams, with 31.4 % for any CT exam and 37.2 % for selective exams only. Depending on the type of exam, between 60 % and 73 % of the respondents read the entire radiology report that they received. I am afraid these data have probably not improved over the years. What it says is that around 40 % of clinicians only value the pictures but do not need the report with it. How often a report is not read and a clinician solely relies on his own interpretation is unknown. In 2000, Margulis and Sunshine [2] wrote about this development which may lead to the risk that clinician specialists may come to believe that radiologists do not contribute sufficiently to the care of patients to justify their presence.

Also, the perception by the general public of the work a radiologist does is very poor. Many think that radiologists are not doctors and have not received medical training. This brings up an important question: what, in the twenty-first century, is the added value of a radiologist for the hospital? Is the radiologist really seen by some as no more than a photographer? I am afraid this is true. Where does this come from? Why is the radiologist often not valued in the same way as specialists in other branches of medicine? To answer this question, we have to go back in history. In the past radiologists often had to work in dark and remote places, for example in the basement of a hospital. You had to know your way in the labyrinth of small corridors to find a radiologist. But, actually, there was no need to find the radiologist, as communication was solely through the request or order forms and the subsequent report. Both clinicians and radiologists were at that time generalists, overseeing their complete—though limited—field of speciality. Diagnostic radiology options were limited in those days and a radiology investigation was often no more than a picture to support or to reject an already standing diagnosis. This world has changed dramatically in recent decades. Clinicians have become subspecialised, with an increasing amount of knowledge about an often small part of medicine. Imaging modalities and options have been revolutionised and have become the most crucial cornerstone in diagnoses and treatment in any hospital. At the same time, demands on the radiologists have grown equally and it is no longer possible to oversee everything and to be an expert in radiology at large. It is also no longer the picture but all the diagnostic image investigations in the context of the whole clinical decision-making that is important. The key element in this now is multidisciplinary communication on the interpretation, not by report or wire, but real communication in patient discussions and teams. Margulis and Sunshine [2] suggested in their millennium paper that teams of radiological subspecialist and clinical specialists should practice together, supporting each other. In 2010, the ESR was still undecided on how enthusiastically subspecialisation in radiology should be promoted [3]. I think the time for hesitation has gone; it is now time to decide on which direction radiology should take in the next decade. Recently, we have seen a fast growth in subspecialty societies within the ESR. There is now also an ESR level III subspecialisation curriculum available.

To me, this points only in one direction: the radiologist should be part of clinical decision-making and be an active clinical partner, with up-to-date clinical knowledge about a medical subspecialty. Many radiologists, however, seem not to have taken this route yet; on the contrary, they still rely on the old report-communication patterns.

But first, let us understand what the key elements of a radiologist actually are:

- To produce medical images
- To report on these images
- To safely store patient information
- To relate images and reporting
- To communicate with clinicians
- To advice on imaging
- To safeguard quality and patient safety

None of these tasks can be taken over by a clinician—at least not in the short term. So, there are opportunities to remain involved, but we now have to change our attitude.

However, some radiologists do not want to stay involved: they have started to leave the clinical environment for teleradiology, moving back to a virtual old remote basement, but now housed in a modern office building outside the hospital. However, only communicating by reports, makes them clinically completely redundant and radiology services are going to be traded as a commodity, based on price. There is no need for clinicians to avoid these radiologists anymore—they have already removed themselves out of the hospital. In the old days, radiologists “owned” and stored the image, which gave them a powerful position; today, every image is instantly available from digital storage. Clinicians have started to learn about the images in their subspecialty territory, and through courses and experience, they have less fear to start making their own interpretations and diagnosis. There is no doubt that the old general radiologist has no added value, maybe only in a group with primary care takers, and will slowly be disappearing in the fog, like old cowboys. But not all is lost; subspecialised radiologists, especially, will have a lot of added value. The radiologist as subspecialised image interpreter, a doctor and a clinical partner, is the future.

The radiologist “version 2.0” should be like an active spider in a web, not like the old radiologist, who could only survive because of the previously mentioned ownership and subsequent storage of the images. I think the analogy with the spider is a good metaphor, because a real spider has a large web and everything that touches his web, even at the edge of the web, is noticed and leads to immediate action by the spider. Usually he then eats what he has caught in the web—something we should not do, of course. But in the spider model, the radiologist should have a network that allows him to notice and detect everything that happens within his subspecialty territory. And the territory should be as big as the subspecialisation of his soulmate, the clinician. And if there is some disturbance, even at the periphery of this network, the radiologist should be pro-active to be present. How to make the step from radiologist to spider? First and foremost, the radiologist 2.0 should be clinically involved. An important step—not just symbolic—is to abolish radiology request forms and replace them with consultancy or advice request forms. Radiology should be leading in imaging and initiate new imaging opportunities, before clinicians ask for it. Radiology should be leading in multidisciplinary teams and organise digital medical rounds at the radiology department. Radiology should set the agenda and timelines for these multidisciplinary teams. The radiology spider should be a teacher, a subspecialty spokesperson, initiator of science and a radiology union person in one. As mentioned before, the radiologist 2.0 should be a doctor and an up-to-date clinical specialist too. The radiologist 2.0 should build clinical-diagnostic units around medical subspecialties (Fig. 1). This means that our future radiology departments should consist of many clinical-diagnostic units. Of course, a radiologist can also be a spider in more than one clinical unit. However, these units should not function as stand-alone units but should be overlapping with cross-fertilisation regarding imaging developments. To achieve this, radiology departments should have their own weekly grand-round and scientific meetings. An internal radiology website could also be very helpful to achieve this. Communication is the magic word, not only with the clinicians but also in your own department. By adopting the spider model with clinical-diagnostic units in the hospital, radiology can stay of added value and at the same token be supportive, but this time not only to the clinician but now also directly to the patient.

**Fig. 1:**
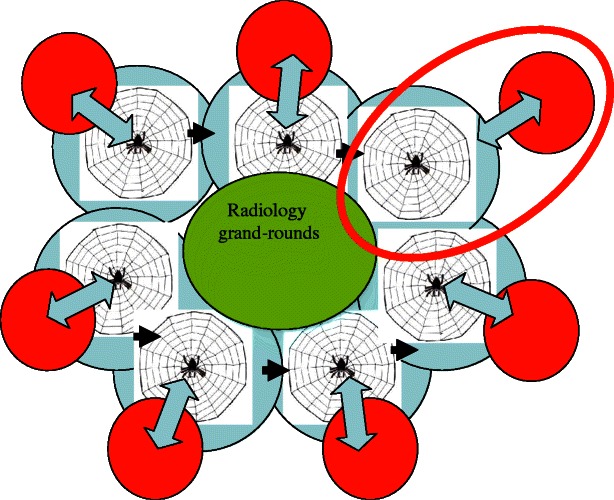
The new spider model. Clinicians (*red discs*) and subspecialised radiologist form clinical-diagnostic units (*red oval*)

I do, however, also realise that by subspecialisation there is a risk for erosion and fragmentation of radiology as a specialty, as pointed out by the ESR executive council in 2009 [4]. But things can and will not stay as they are today. We should take the future in our own hands and not start acting like an ostrich. We have this obligation to our young colleagues who are entering radiology today. Only if we start building these new departments today will radiology still have a glorious future. However, if we outsource ourselves, I have serious doubts about our future.

Of course there should be coordination and guidance to make this change possible. Here I see an important task for the ESR [3]. The ESR has already produced a template for subspecialty training (Level III) and certification and diplomas through the European Board of Radiology. Also, the ESR is offering a broad spectrum of high-level subspecialty training opportunities through ESOR. ESR members can also participate in subspecialty training offered by the various subspecialty societies, like the European School of Interventional Radiology (ESIR) organised by CIRSE. Subspecialty societies, under the leadership of the ESR, should get more and more involved in the future of radiology. We need a strong organisation but we also need involved ambassadors for radiology at the various subspecialty areas.
